# Visual feedback increases expiratory muscle training effort in Parkinson’s disease: a randomised crossover trial

**DOI:** 10.1186/s12984-026-02104-5

**Published:** 2026-07-22

**Authors:** Martin Srp, Katerina Dvorakova, Jan Muzik, Petra Janderova, Martin Sevcik, Vaclav Matys, Jakub Vitek, Radim Kliment, Martina Hoskovcova, Evzen Ruzicka

**Affiliations:** 1 Department of Neurology, Centre of Clinical Neuroscience, First Faculty of Medicine, General University Hospital, Charles University, Katerinska 30, Prague 2, 128 21 Prague, Czech Republic; 2 Department of Information and Communication Technologies in Medicine, Faculty of Biomedical Engineering, Czech Technical University, Prague, Czech Republic; 3 Institute of Thermomechanics, Czech Academy of Sciences, Prague, Czech Republic

**Keywords:** Parkinson’s disease, Expiratory muscle strength training, mHealth, Visual feedback

## Abstract

**Background:**

Disorders of airway protection, including impaired cough and swallowing, are clinically important complications of Parkinson’s disease. Expiratory muscle strength training (EMST) is an established non-pharmacological intervention targeting these deficits. The aim of this study was to evaluate the acute effect of visual feedback provided by the SpiroGym mHealth app on expiratory muscle activation during EMST in people with Parkinson’s disease.

**Methods:**

We conducted a single-center, randomised, two-period crossover trial in people with Parkinson’s disease. Participants performed EMST under two conditions, in randomised order: conventional EMST without visual feedback and EMST with real-time visual feedback provided by the SpiroGym app. Expiratory muscle activation was measured using surface electromyography of the abdominal muscles. The primary outcome was the within-participant difference in mean peak normalised internal oblique activity between conditions. Secondary outcomes included external oblique and rectus abdominis activity, associations with neuropsychiatric symptoms, and participant-reported usability ratings.

**Results:**

Twenty participants were randomised, and 19 completed the study and were included in the final analysis. No study-related adverse events occurred. Visual feedback significantly increased mean peak normalised internal oblique activity compared with no feedback (118.2 ± 36.4% vs. 90.5 ± 23.0% of reference voluntary contraction; *p* < 0.001; Cohen d = 0.95). Without feedback, internal oblique activation significantly declined from 100.1 ± 22.2% in the first set to 84.4 ± 25.5% in the fifth set, whereas it remained stable with feedback (120.3 ± 29.4% to 119.9 ± 40.6%). External oblique activation was also significantly higher with feedback (122.2 ± 38.1% vs. 100.9 ± 32.4%; *p* = 0.002; Cohen d = 0.66), while rectus abdominis activation did not differ significantly between conditions (105.2 ± 41.3% vs. 90.2 ± 36.1%; *p* = 0.06; Cohen d = 0.43). No significant associations between feedback responsiveness and depression, apathy, or anxiety were detected. Participants rated the visual feedback as easy to understand and helpful for EMST performance.

**Conclusions:**

Real-time visual feedback delivered by the SpiroGym app acutely increased and stabilized expiratory muscle activation during EMST in people with Parkinson’s disease.

*Trial registration* ClinicalTrials.gov NCT06942533, 1 May 2025.

**Supplementary Information:**

The online version contains supplementary material available at https://doi.org/10.1186/s12984-026-02104-5.

## Background

 Parkinson’s disease (PD) is one of the fastest growing neurodegenerative disorders worldwide. This trend is partly driven by population ageing, as the incidence of PD rises with age [1]. However, age-adjusted prevalence is also increasing, suggesting additional contributors such as improved diagnostic accuracy and environmental factors [2]. Resistance training plays an important role in the non-pharmacological management of PD [3]. Such interventions may target several characteristic PD-related impairments, including bradykinesia, reduced muscle strength, impaired balance, gait difficulties and reduced functional mobility. In addition, resistance training may have beneficial effects on selected non-motor domains, including cognition and quality of life [4, 5]. They have also been associated with slower disease progression [6] and improved quality of life [5]. One PD-specific application of resistance training aimed at supporting airway protection is expiratory muscle strength training (EMST) [7]. Airway protection is clinically relevant in PD, as both cough and swallowing are frequently impaired. These deficits are multifactorial and may involve impaired muscle control and peripheral muscle weakness [8, 9]. This impairment increases the risk of aspiration and aspiration pneumonia is a leading cause of death in people with PD (PwP) [10–12]. EMST is performed using a calibrated device with a one-way, spring-loaded valve that provides resistance during forced expiration, thereby overloading the expiratory muscles involved in cough and the suprahyoid muscles involved in swallowing, and improving their force-generating capacity [9, 13]. This provides a rationale for targeting the muscle weakness component that may contribute to cough and swallowing dysfunction. In addition, because the EMST maneuver mimics many of the movements required for cough production, it may also address the impaired coordination component [14].

Available detraining data indicate that EMST-related gains may be partly retained after intensive training [13]. Other research, by contrast, found that detraining was more pronounced in individuals with poorer baseline swallowing function, in whom a three-month detraining period returned swallowing function to baseline (pre-EMST) levels [15]. This suggests that, in PwP receiving EMST for clinically relevant cough or swallowing impairment, maintenance programmes are needed to sustain the functional gains [15, 16]. However, the optimal duration, frequency, and intensity of long-term EMST in PwP remain insufficiently established in the current literature. In this context, SpiroGym, a mobile health (mHealth) application designed to support long term adherence to EMST by providing real-time visual feedback and longitudinal tracking of training progress, has been introduced [17–19]. In a previous randomised controlled trial, PwP were randomised to conventional EMST or the same EMST protocol enhanced with the SpiroGym app [20]. The study protocol consisted of 8 weeks of semisupervised intensive EMST followed by 16 weeks of unsupervised maintenance training. One key finding was that expiratory muscle strength, measured as maximal expiratory pressure (MEP), improved in both groups, but the gains were significantly larger in the SpiroGym group at the 8-week visit. Because the intensity of EMST was identical in both groups and adherence rates were comparable, the greater strength gains observed with SpiroGym may reflect more effective within-session training effort facilitated by real-time visual feedback [20]. This interpretation is consistent with evidence from resistance training research showing that visual feedback can enhance acute performance within a training session and promote greater long-term adaptations [21]. However, it remains unclear whether visual feedback increases acute expiratory effort during repeated EMST maneuvers in PwP.

Therefore, the primary objective of this randomised crossover trial was to evaluate the acute effect of visual feedback provided by the SpiroGym app on expiratory muscle activation during EMST in PwP, as assessed by surface electromyography (sEMG). We hypothesized that visual feedback provided by the SpiroGym app would increase expiratory muscle effort during EMST compared with EMST performed without feedback.

## Methods

### Study design

This was a two-period, two-sequence (AB/BA), single-center, randomised crossover trial, with each participant receiving both interventions: a control condition (EMST without visual feedback) and an experimental condition (EMST with visual feedback). The rationale for the crossover design was to enable each participant to serve as their own control, thereby reducing the impact of interparticipant variability on study outcomes, minimizing confounding factors, and enabling a more precise comparison of the effects of visual feedback.

The trial was prospectively registered (ClinicalTrials.gov NCT06942533; 1 May 2025) and reported in accordance with CONSORT guidelines for randomised crossover trials [22]. The intended primary outcome was internal oblique (IO) muscle activation during EMST, quantified by surface electromyography (sEMG), with external oblique (EO) and rectus abdominis (RA) activation as secondary outcomes. In the original registration, however, these outcomes were specified at the group level (“abdominal muscles”) rather than at the level of individual muscles. The registration was subsequently updated on 24 November 2025, before the statistical analysis, to name the IO as the primary outcome and the EO and RA as secondary outcomes. This refinement affected only the labelling of the outcomes in the public registry and did not change the hierarchy of outcomes or how they were recorded, analysed, or reported. The trial was conducted at the Department of Neurology, General University Hospital, First Faculty of Medicine, Charles University in Prague, Czech Republic, between May 2025 and November 2025.

### Participants

Participants were recruited through convenience sampling from the Movement Disorders Centre at the study site between May 2025 and September 2025. Inclusion criteria were: (1) a diagnosis of PD confirmed by a movement disorder specialist according to the UK Brain Bank criteria [23], (2) modified Hoehn and Yahr stage of I–IV in the ON medication state, (3) age between 40 and 80 years, and (4) a stable dopaminergic medication dose for ≥ 1 month prior to enrollment and throughout the study period. Exclusion criteria were: (1) suspected parkinsonism due to causes other than idiopathic PD, (2) significant cognitive impairment (Montreal Cognitive Assessment score < 19) [24], (3) severe respiratory disease documented in the medical record, (4) uncontrolled hypertension, (5) severe visual impairment not correctable with lenses; and (6) previous experience with EMST and with the SpiroGym app.

### Randomisation and blinding

A simple computer-generated random allocation sequence was created prior to study initiation using a web-based randomisation tool (randomizer.org) by the study team. Participants were randomised in a 1:1 ratio to one of two treatment sequences (Control-Experimental, or Experimental-Control). Allocation concealment was ensured by using sealed opaque envelopes containing the sequence order, which were opened only after the participant had been enrolled in the study. Assessor blinding was not feasible during data collection because the use of the SpiroGym app in the experimental condition was apparent. However, sEMG data were de-identified and condition-coded after acquisition, and the assessor extracting sEMG outcomes was blinded to condition. The study statistician was blinded to experimental condition and analyzed condition-coded data. Participants were informed that the study compared the acute effects of two EMST conditions and that they would complete both conditions in a randomised order. They were not provided with a specific study hypothesis. If participants asked which condition was expected to be more effective, they were told that both were considered beneficial and that the study aimed to evaluate their effects.

### Study outcome parameters

The primary outcome was the within-participant difference in mean peak normalised sEMG activity of the IO muscle between EMST performed with and without visual feedback. The IO was selected as the primary outcome for three reasons. First, abdominal muscles as a group are key contributors to forced expiratory effort [25, 26], with significantly greater contributions from the IO and transversus abdominis (TA) than from the EO and RA [26, 27]. Second, the TA lies deep to the IO and EO abdominal muscles, and therefore cannot be selectively assessed using sEMG. Third, EMST in the present study was performed at 75% of MEP. At this intensity, the IO has been reported to be the only abdominal muscle reaching an activation level (> 40% of maximal voluntary contraction) considered sufficient to drive strength-related adaptations, whereas the EO and RA remain below this level [28]. Accordingly, IO sEMG during EMST was used as a physiological surrogate of within-session expiratory effort that may help explain the previously observed between-group differences in MEP [20]. Prespecified secondary outcomes included within-participant differences in mean peak normalised EO and RA sEMG activity to characterize the pattern of superficial abdominal muscle recruitment during EMST and participant-reported subjective ratings of SpiroGym usability, perceived visual feedback helpfulness, and sEMG electrode-related discomfort. Additional exploratory outcomes included correlations between the within-participant difference in normalised IO sEMG activation and self-reported neuropsychiatric symptoms assessed using questionnaires validated for use in Parkinson’s disease: the Beck Depression Inventory-II (BDI) [29] for depression, the Starkstein Apathy Scale (SAS) [30] for apathy, the State-Trait Anxiety Inventory for anxiety state (STAI-state) and trait (STAI-trait) [31].

### Assessment visits

**Fig. 1:**
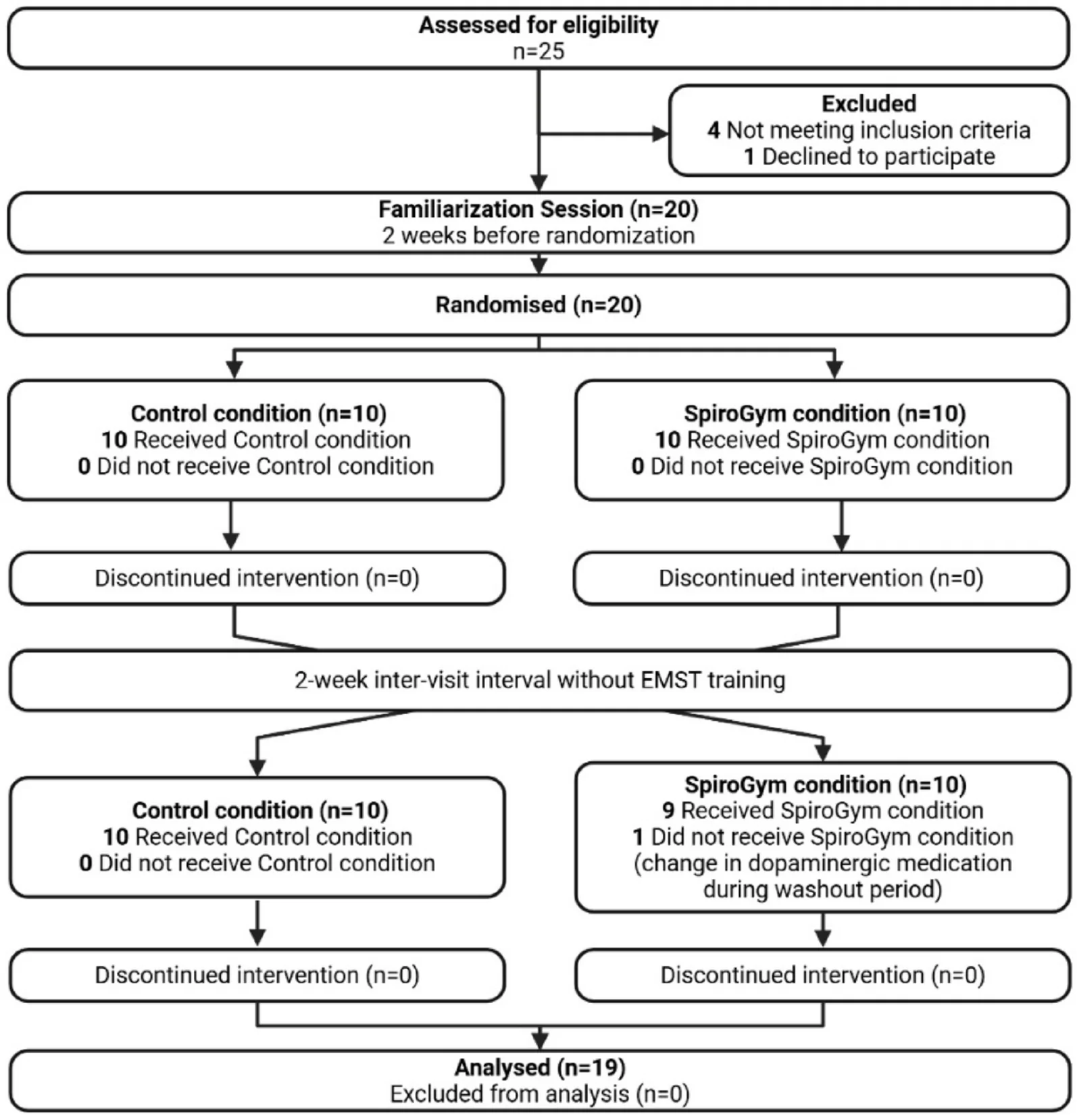
The CONSORT flow diagram

All assessments were conducted in the ON medication state (approximately 1 h after intake of antiparkinsonian medication) and at the same time of day. For the study flow diagram, see Fig. 1. The two study visits were separated by a two-week interval without EMST training to minimize potential carryover effects. This interval was chosen because neural adaptations that can occur after a single session of resistance training, and could otherwise influence between-visit differences in sEMG, are expected to dissipate over a two-week rest period [32]. PwP were instructed to keep their physical activity as similar as possible during the two days preceding each study visit in order to minimize potential differences in fatigue. During both the control and experimental conditions, the study team did not encourage or motivate participants. Instead, instructions were delivered in a neutral tone, using only “pause” (to indicate that EMST should not be performed) and “now” (to cue the participant to perform the EMST maneuver). Before each study visit, PwP were assessed for fatigue using the visual analog fatigue scale (VAFS; 0 = no fatigue, 10 = worst possible fatigue). The VAFS has been shown to be a reliable measure of overall fatigue in individuals with PD [33]. In addition, to reduce potential learning effects, i.e. that PwP would perform EMST better after the first training condition, a practice (familiarisation) session was held two weeks before the main experiment. During this session, MEP was assessed using a flanged rubber mouthpiece connected to a pressure manometer (MicroRPM, Vyaire Medical), in accordance with current recommendations [34]. Participants were then introduced to EMST and practiced the maneuvers without visual feedback at 75% of their MEP — the same resistance subsequently used in both the control and experimental conditions. Practice continued until the study physiotherapist deemed the technique and maneuver quality satisfactory. MEP was measured during the familiarisation session only and was not re-measured at the first or second study visit. The EMST150 load used in both conditions was therefore set at 75% of the MEP obtained during familiarisation. Finally, neuropsychiatric symptoms were self-assessed on the day of the familiarisation session.

### sEMG

sEMG data were collected using the BTS FreeEMG 1000 (BTS Bioengineering, Italy) with a sampling frequency of 1000 Hz. Prior to sensor attachment, hair at each electrode site was removed and the skin was gently abraded and cleansed to reduce impedance, then allowed to dry [35]. The wireless sensors were attached to the skin using Ag/AgCl surface electrodes (Kendall™ H124SG, Cardinal Health UK, Leeds) with an inter-electrode distance of 20 mm. The sensors were securely fixed with additional tape to prevent movement artifacts. Electrodes were positioned over the muscle belly, between the innervation zone and the tendon, following the recommendations [36] for IO and EO and [37] for RA. For the IO, the electrode pair was placed 2 cm below the most prominent point of the anterior superior iliac spine, just medial and superior to the inguinal ligament, lateral to the innervation zone. For the EO, the electrode pair was placed 14 cm from the midline, just below the level 1 cm above the umbilicus and at least 1 cm above the iliac crest. The electrodes for the RA were placed on the lower abdominal wall, 2 cm lateral and caudal to the umbilicus. We placed the sensors unilaterally over the IO, EO and RA muscles (always on the right side). Unilateral placement was chosen because previous EMG studies have reported no significant mean differences in muscle activity between the more and less affected sides in PwP [38], and to reduce examination time and participant burden. During each sEMG examination, the assessor monitored for the presence of levodopa-induced dyskinesia, which could affect the sEMG recordings [39]. If clinically relevant dyskinesia occurred, the assessment was postponed until the dyskinesia had subsided to avoid contamination of the sEMG recordings. In addition, all raw sEMG recordings were visually inspected for electrocardiographic (ECG)-related artifacts [40]. The raw signals were first filtered with a Butterworth high-pass filter (cutoff 20 Hz) and a Butterworth low-pass filter (cutoff 450 Hz), and then full-wave rectified. A 50 ms moving root mean square window was applied to obtain a smoothed signal. Peak amplitude for each expiratory maneuver was identified and extracted from anonymized, condition-coded sEMG files by an assessor blinded to the intervention condition. All signals were normalised to the subject reference voluntary contraction (RVC), as used in previous research in PwP [41]. Maximal voluntary contraction (MVC) was not employed, because recommended MVC protocols [37] for the abdominal muscles (e.g. side bridge and prone bridge) are not feasible for many PwP. To obtain the RVC, participants performed maximal voluntary cough maneuvers. During maximal voluntary coughing, all abdominal muscles are strongly activated as forced expiratory muscles [42]. RVC measurements were performed before each EMST study visit. The RVC obtained at each study visit was used to normalise the EMST sEMG signals recorded during that same visit. The RVC was used as a reproducible reference task for sEMG normalisation rather than as a maximal ceiling. Therefore, EMST performed against 75% of MEP could produce peak sEMG amplitudes exceeding those recorded during a single voluntary cough. Values above 100% RVC were thus expected and did not indicate supramaximal effort. Participants sat comfortably in a chair, and sEMG was recorded simultaneously from the IO, EO, and RA. Once the sEMG signal had stabilized, participants were instructed to “cough as hard as possible, as if something went down the wrong pipe.” The same cough trials were used to derive the RVC for all recorded muscles (IO, EO, and RA). Each participant performed three voluntary cough trials, separated by a 1-minute rest. For each trial, the sEMG signal was analysed to determine the peak amplitude. For each muscle, the mean of the three peak amplitudes was then calculated and used as the RVC for normalising EMG data.

### Participant-reported outcomes

After completing both study visits, participants completed a questionnaire consisting of three items assessing their impressions of performing EMST with and without visual feedback. Specifically, the questionnaire evaluated (1) ease of understanding the information displayed in the SpiroGym app, (2) perceived helpfulness of SpiroGym visual feedback for EMST performance, and (3) discomfort related to the sEMG electrodes during EMST. Responses were recorded on 5-point Likert scales. The questionnaire was inspired by previously published questionnaires [41, 43] and was adapted for the purposes of the present study.

### Training protocols

#### Control condition

Participants performed a conventional EMST protocol [9] consisting of five sets of five forceful expirations using an expiratory muscle trainer (the EMST150; Aspire Products, LLC, United States). The EMST150 load was set at 75% of each participant’s MEP. Seated upright, participants were instructed to occlude their nose with nose clips, take a big breath in, and blow as forcefully as possible into the EMST150 to open the valve. The rest interval between expiratory maneuvers within a set was 15 s, and the rest interval between sets was 1 min. Before the start of measurements, each participant performed two EMST maneuvers to verify that they understood the task. The EMST150 device provided to participants for the purposes of the study was collected and stored in the examiner’s office after completion of the first study visit, to ensure that participants could not practice at home before the second study visit.

#### Experimental condition

**Fig. 2:**
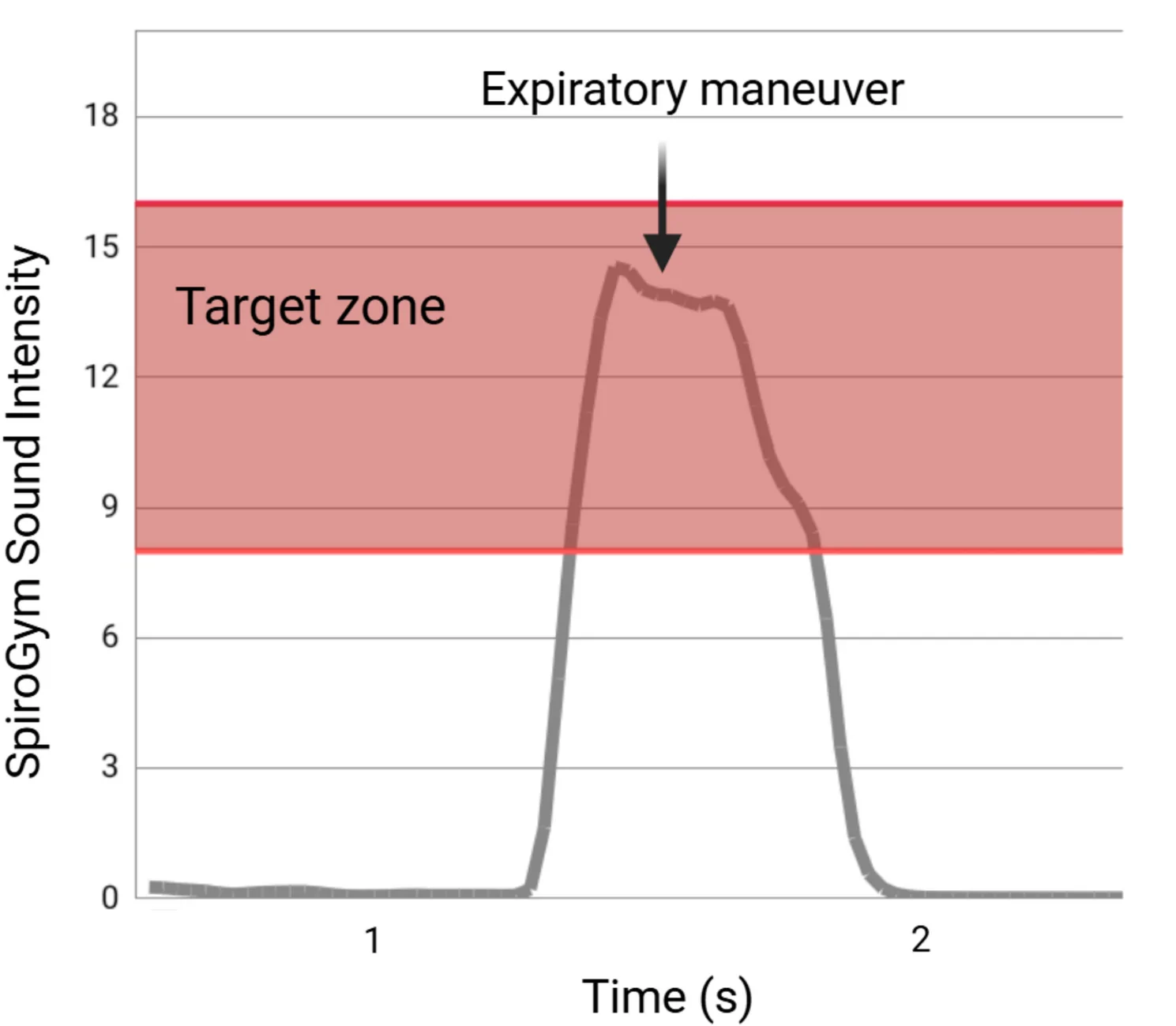
Example of the real-time SpiroGym visual feedback display during a single expiratory maneuver. Participants were instructed to take a deep breath in and blow as forcefully as possible to open the valve, using the real-time SpiroGym curve (grey trace) as feedback to raise the SpiroGym Sound Intensity as high as possible. The highlighted band (red) indicates the target zone corresponding to the prescribed expiratory effort

Participants followed the same EMST protocol as in the control condition, but the EMST150 was coupled with the *SpiroGym* app on a study smartphone (Samsung Galaxy A20e). The SpiroGym app monitors expiratory training in real time by analysing acoustic signals captured via an external microphone (Speed Link; SL-8691-SBK-01) mounted on the EMST device using a custom 3D-printed ring fabricated by our team. This attachment does not alter resistance, airflow, or valve function and standardizes the microphone’s position and angle. The microphone was connected to the smartphone via a 3.5-mm jack. When the training is performed correctly, the valve of the EMST150 opens, generating increased airflow and sound. These signals are converted by the app into a visual feedback curve displayed on the smartphone (Fig. 2). Full technical details of the digital signal processing have been reported previously [19]. The study team positioned the smartphone at the participant’s eye level, and each participant adjusted the viewing distance (approximately 30–60 cm) to ensure optimal visibility of the SpiroGym feedback screen. Participants were familiarised by the study team with the visual feedback provided by the SpiroGym app. Participants were instructed to take a deep breath in and blow as forcefully as possible to open the valve, using the SpiroGym curve as real-time feedback to try to raise it as high as possible. Each participant performed two EMST maneuvers with visual feedback before measurements began.

#### Harms

In accordance with CONSORT Harms recommendations, adverse events were collected systematically at each visit by the study team [44]. Participants were asked an open-ended question (“Did you experience any unpleasant symptoms during or after the session?”) followed by a prespecified checklist (e.g., dizziness/vertigo, shortness of breath, headache, chest discomfort, nausea, excessive fatigue, syncope). Adverse events were recorded for the period during the session and for 15 min thereafter. In addition, any adverse events occurring between visits were captured at the subsequent visit.

#### Sample size

Sample size calculations were based on the primary outcome. Because no prior studies had compared IO sEMG during EMST with versus without visual feedback, we estimated the effect size using an internal pilot analysis. The sample size for this analysis was determined according to the recommendations [45]. The internal pilot analysis included 12 participants who were subsequently retained in the final analysed sample. This analysis suggested a 39% between-condition difference in mean peak normalised IO sEMG activity, with an estimated effect size of Cohen d = 1.095. A power analysis using G*Power 3.1.9.3 [46] indicated that a total sample size of 13 participants would provide 95% power to detect this effect size with a two-sided paired t-test at an alpha level of 0.05. To enhance the robustness of our findings, we aimed to include 20 participants.

### Statistical analysis

All analyses were conducted in MATLAB R2025b (MathWorks, Natick, MA, USA). Because the data did not meet the assumptions of normality, all comparisons used the two-sided Wilcoxon signed-rank test. For the IO (the primary outcome), both within-condition set-wise comparisons (each of Sets 2–5 versus Set 1) and between-condition comparisons (FB versus No-FB) were performed. For the EO and RA, only between-condition comparisons (FB versus No-FB) were performed. To account for multiplicity, p-values were adjusted using the step-down Bonferroni–Holm method (two-sided α = 0.05) within three separate families: the within-condition set-wise comparisons in the No-FB condition (4 comparisons), the within-condition set-wise comparisons in the FB condition (4 comparisons), and the between-condition comparisons across the five sets (5 comparisons). Both raw and adjusted p-values are reported. Adjusted p-values are capped at 1.000. Other secondary outcomes were exploratory and were not adjusted for multiple comparisons. Any apparent significance among these should be confirmed in future research. Effect sizes for between-condition differences were expressed as Cohen d with 95% confidence intervals, interpreted as moderate (≥ 0.5) or large (≥ 0.8). Cohen d was computed from the standard deviation of the paired difference scores (d_z), and its 95% confidence interval was obtained by bootstrap resampling, using the meanEffectSize function in MATLAB R2025b. The median difference between conditions was estimated using the Hodges–Lehmann estimator with a 95% confidence interval. VAFS scores and sEMG activity by randomisation arm were compared between conditions using the two-sided Wilcoxon signed-rank test. Associations between the within-participant difference in normalised IO sEMG activation and the BDI, SAS, and STAI scores were examined using Spearman’s rank correlation coefficient. Inter-rater reliability of sEMG data extraction was assessed by having two raters, blinded to the experimental conditions, independently analyse all sEMG data from five randomly selected participants (26% of all data). Agreement was quantified using an intraclass correlation coefficient (ICC 2,1). The robustness of the primary outcome to non-normalisation was examined by reanalysing the within-participant difference in mean peak (non-normalised) IO sEMG activity using the two-sided Wilcoxon signed-rank test. Separately, crossover-specific assumptions were assessed using a generalised linear mixed-effects model with a Gamma distribution and a reciprocal link, which expresses the model coefficients on the 1/%RVC scale. The model included fixed effects for condition, sequence, and their interaction, and a random intercept for participant. In this AB/BA design, the sequence × condition interaction corresponds to the carryover effect. Statistical significance was set at *p* < 0.05. No interim analyses were performed except for sample-size estimation.

### Ethical considerations

This study was conducted in accordance with the principles of the Declaration of Helsinki. The study was approved by the Ethics Committee of the General University Hospital in Prague (protocol number Z 63/25 S-IV AP). All participants provided written informed consent prior to any study procedures, including consent for collection, analysis, and publication of de-identified data. Participants did not receive any financial compensation. All data were de-identified before analysis and stored securely in accordance with all applicable data protection regulations and institutional data protection policies.

## Results

Twenty participants who met the inclusion criteria received the interventions in a randomised order. Table 1 presents the demographic and clinical characteristics of included participants. One participant was withdrawn due to a change in dopaminergic medication during the study period (Table 2). Thus, 19 participants were included in the final analysis. No study-related adverse events occurred, and participants reported no discomfort from the sEMG electrodes during EMST (Table 3). Inter-rater reliability for sEMG data extraction was excellent, with an ICC (2,1) of 1.00 (95% CI: 0.99–1.00). The intra-session reliability of peak sEMG amplitudes across the three voluntary cough trials for IO, EO and RA was good to excellent and is reported in Supplementary Table (1) Visit-1 and visit-2 peak sEMG amplitudes from the voluntary cough trials (mean of three trials) for each muscle are presented in Supplementary Table (2) Pre-session VAFS scores did not differ significantly between conditions (*p* = 0.514; mean ± SD with feedback: 3.00 ± 1.53; without feedback: 2.63 ± 2.31). There were no missing data for the primary or secondary analyses. No participant exhibited levodopa-induced dyskinesia during either EMST session, and visual inspection of the raw sEMG recordings identified no relevant ECG-related artifacts likely to affect peak amplitude extraction.

**Table 1:** Baseline demographic and clinical characteristics overall and by randomisation arm

Characteristic	Total (*n* = 20)	Control–SpiroGym (*n* = 10)	SpiroGym–Control (*n* = 10)
Age (years)	56 (50–64.5)	61.5 (55–70)	53 (49–60)
Gender (male/female)	10/10	5/5	5/5
Weight (kg)	73 (67.5–90)	71 (65–79)	88.5 (72–92)
Height (cm)	176.5 (172–180)	175.5 (167–177)	178.5 (176–188)
Disease duration (years)	5 (2.5–6.5)	5.5 (2–6)	4.5 (3–7)
UPDRS III, ON state	5.5 (4.5–9)	6.5 (5–9)	5 (3–6)
Hoehn & Yahr stage	1 (1–2)	1.5 (1–2)	1 (1–2)
LEDD (mg/day)	600 (450–875)	705 (450–1700)	530 (450–765)
MoCA	26.5 (25.5–27.5)	26 (24–27)	27 (26–28)
MEP (cmH_2_O)	158 (112.5–176.5)	112.5 (86–163)	172 (153–190)
STAI State	39.5 (27.5–48)	40.5 (32–52)	34.5 (25–44)
STAI Trait	41 (24–58)	45.5 (31–48)	33 (26–44)
BDI-II	8 (4–13.5)	10 (5–18)	8 (3–10)
SAS	8 (3–13.5)	8 (7–15)	7 (3–12)

Values are Median (Interquartile range). *MoCA* Montreal Cognitive Assessment, *LEDD* Levodopa Equivalent Daily Dose, *MDS-UPDRS III* Unified Parkinson’s Disease Rating Scale motor score, *MEP* Maximum expiratory pressure, *STAI* The State-Trait Anxiety Inventory, *BDI-II* Beck Depression Inventory, *SAS* Starkstein Apathy Scale

**Table 2:** Set-wise internal oblique sEMG activation (% RVC) during EMST with (FB) and without (No-FB) visual feedback

Set	No-FB mean ± SD	FB mean ± SD	Within-condition (*p* vs. Set 1)		Between-condition (FB vs. No-FB)	
			No-FB, raw (adj)	FB, raw (adj)	*p*, raw (adj)	Cohen d [95% CI]
1st	100.1 ± 22.2	120.3 ± 29.4	–	–	0.006 (0.006)	0.76 [0.11, 1.40]
2nd	92.1 ± 23.4	120.3 ± 38.2	0.020 (0.024)	0.573 (1.000)	0.001 (0.004)	0.87 [0.21, 1.52]
3rd	87.5 ± 19.0	113.8 ± 31.9	0.002 (0.007)	0.049 (0.194)	0.002 (0.004)	0.98 [0.31, 1.64]
4th	88.2 ± 24.8	116.8 ± 41.7	0.003 (0.006)	0.334 (1.000)	0.001 (0.004)	0.82 [0.16, 1.46]
5th	84.4 ± 25.5	119.9 ± 40.6	0.002 (0.007)	0.658 (1.000)	0.001 (0.004)	1.03 [0.35, 1.68]

Values are mean ± standard deviation of normalised peak IO sEMG activation (% RVC). All comparisons used two-sided Wilcoxon signed-rank tests; p-values are shown as raw (Bonferroni–Holm-adjusted). Within-condition comparisons test each of Sets 2–5 against Set 1, adjusted within each condition separately (4 comparisons per condition). Between-condition comparisons (FB vs. No-FB) were adjusted within a single family across the five sets (5 comparisons). Adjusted p-values are capped at 1.000. Cohen d (with 95% confidence interval) refers to the between-condition difference at each set. FB, visual feedback; No-FB, no visual feedback; RVC, reference voluntary contraction

**Table 3:** Participant reported questionnaire

Question	Response	% (*n*)
Difficulty understanding the information displayed in the SpiroGym app’s visual feedback	Very difficult	0 (0)
	Difficult	0 (0)
	Neutral	0 (0)
	Easy	0 (0)
	Very easy	100 (19)
Degree to which SpiroGym feedback helps EMST performance*	Not helpful at all	0 (0)
	Not very helpful	5.3 (1)
	Neutral	5.3 (1)
	Helpful	21.1 (4)
	Very helpful	68.4 (13)
Did you experience discomfort from the sEMG electrodes during EMST?	No discomfort at all	100 (19)
	Slight discomfort	0 (0)
	Neutral	0 (0)
	Moderate discomfort	0 (0)
	Extreme discomfort	0 (0)

* Participants were also asked an open-ended question: “Please specify.” The responses can be found in Supplementary Table 7

### Primary outcome

**Fig. 3:**
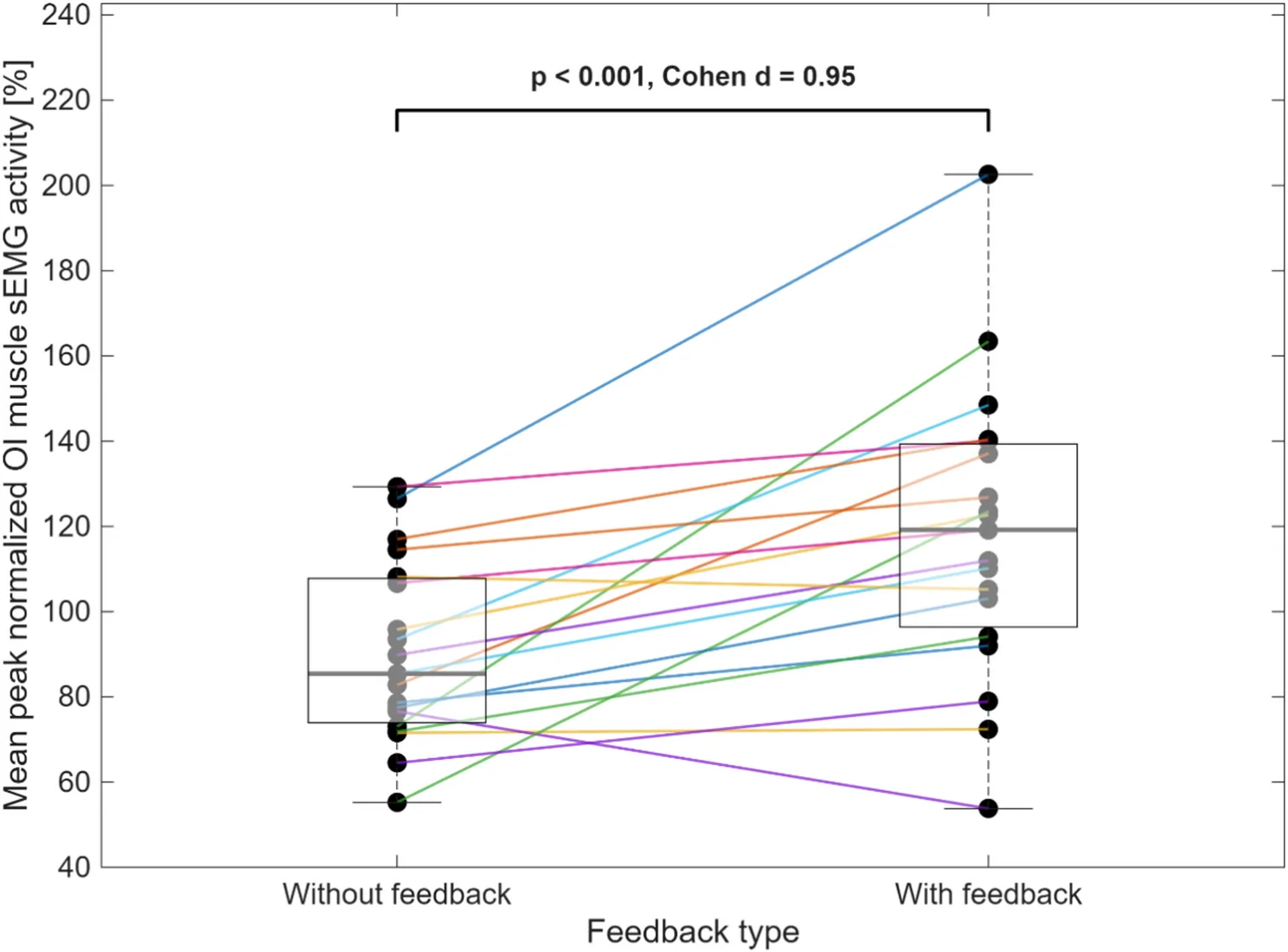
Individual and group-level differences in mean peak normalised internal oblique muscle sEMG activity during EMST performed with and without visual feedback

The within-participant difference in mean peak normalised IO sEMG activity during EMST was significantly greater with visual feedback than without (%RVC with feedback: 118.2 ± 36.4; without feedback: 90.5 ± 23.0; Cohen d = 0.95 [95% CI 0.29, 1.61]; *p* < 0.001; Fig. 3). In non-parametric terms, the median values were 116.8%RVC (IQR 94.1–139.9) with feedback and 84.6%RVC (IQR 74.5–108.6) without feedback (Wilcoxon W = 166), and the Hodges–Lehmann estimator of the median paired difference (feedback − no feedback) was 23.7%RVC (95% CI 12.9–40.9).

A non-normalised sensitivity analysis, conducted to evaluate the robustness of the primary outcome, likewise showed significantly greater mean IO sEMG activity with visual feedback (raw amplitude with feedback: 0.084 ± 0.062 mV; without feedback: 0.056 ± 0.042 mV; Cohen d = 0.48 [95% CI 0.16, 1.11]; *p* < 0.001; Supplementary Table 3). In the mixed-effects model, the effect of visual feedback remained significant (estimate = − 0.0027, 95% CI [− 0.0050, − 0.0003], *p* = 0.026), with no significant sequence effect (*p* = 0.772) or sequence × condition interaction (*p* = 0.933). The model coefficient is on the reciprocal (1/%RVC) scale and is therefore not directly comparable to the descriptive difference. On the %RVC scale the model estimated about 92%RVC without feedback and 122%RVC with feedback. For results from the mixed-effects model see Supplementary Table 4. Because the interaction term is included, the condition coefficient represents the simple effect within the reference sequence rather than the pooled effect, which contributes to the larger p-value (*p* = 0.026) relative to the primary within-participant comparison (*p* < 0.001). Mean normalised IO sEMG activity results by randomization arm are provided in Supplementary Table 5.

**Fig. 4:**
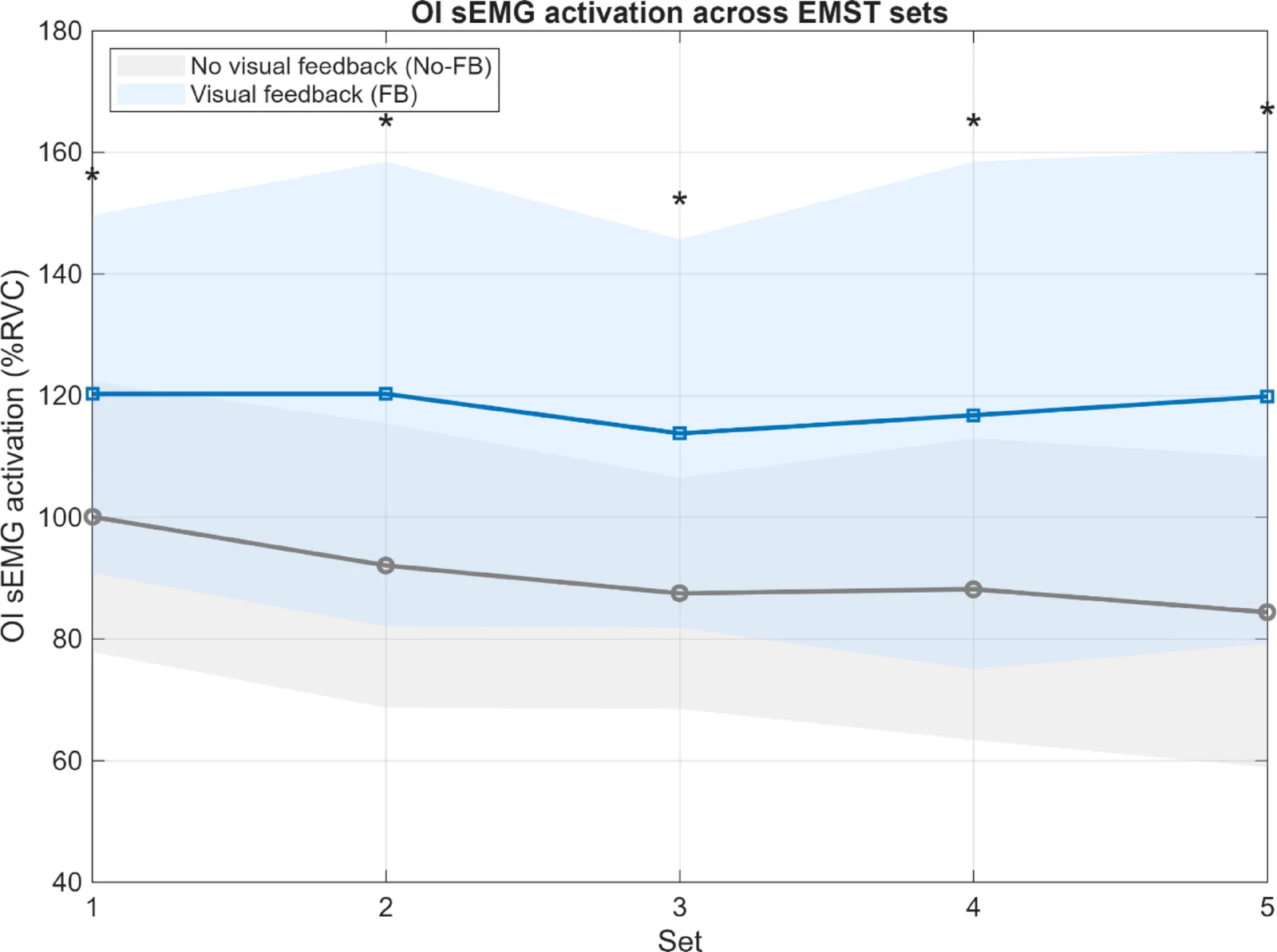
Mean peak normalised internal oblique sEMG activity across five EMST sets in the no-visual-feedback and visual-feedback conditions. Lines represent mean values, and shaded bands indicate standard deviations. Asterisks indicate statistically significant between-condition differences at individual sets (adjusted *p* < 0.01)

Set-wise comparisons showed higher normalised IO sEMG activity with visual feedback in all sets (adjusted *p* = 0.004–0.006; Table 2; Fig. 4). In the no-feedback condition, IO sEMG activation declined significantly from the first set across sets 2–5 (all adjusted *p* ≤ 0.024), whereas no significant differences were observed across sets 1–5 with visual feedback (all adjusted *p* > 0.19; Table 2).

### Secondary outcomes

The within-participant difference in mean peak normalised EO sEMG activity during EMST performed with visual feedback was significantly greater than without feedback (%RVC with feedback: 122.2 ± 38.1; without feedback: 100.9 ± 32.4; Cohen *d* = 0.66 [95% CI 0.01, 1.3]; *p* = 0.002). No difference was observed in RA sEMG activity with and without visual feedback (%RVC with feedback: 105.2 ± 41.3; without feedback: 90.2 ± 36.1; Cohen d = 0.43 [95% CI -0.2, 1.06]; *p* = 0.06). No significant correlations (all *p* ≥ 0.449) were observed between the difference in normalised IO sEMG activation between EMST with and without visual feedback and BDI, SAS, or STAI (Supplementary Table 6). Table 3 summarizes participants’ reported outcomes.

## Discussion

This randomised crossover study examined the acute effects of visual feedback provided by the SpiroGym app on expiratory muscle activation during EMST in PwP. The main finding was that visual feedback resulted in significantly higher IO sEMG activation during expiratory maneuvers compared with EMST performed without feedback. Importantly, this effect was consistent across all training sets. In contrast, when visual feedback was absent, IO activation declined significantly after the first set, indicating a progressive reduction in expiratory effort within the training session. Our observation that SpiroGym visual feedback resulted in 31% higher IO sEMG activation compared with the no-feedback condition aligns with a recent meta-analysis showing that visual feedback is superior to no feedback for acute resistance training performance [21]. A commonly proposed mechanism is that visual feedback enhances motivation and competitiveness, thereby promoting greater effort during task execution [47–49]. This interpretation is consistent with our participants’ open-ended responses, as motivation was the most frequently reported perceived benefit of SpiroGym visual feedback. Another plausible mechanism through which SpiroGym feedback may influence EMST performance is through attentional focus. Attentional focus refers to where an individual directs their attention during task execution (internal focus vs. external focus) [50]. In the SpiroGym condition, the real-time visualization of the achieved outcome encourages an external focus (focusing on the SpiroGym visual feedback), whereas, without visual feedback, attentional focus may be directed more toward internal cues related to breathing mechanics. Evidence from a recent meta-analysis in resistance training indicates that coaching an external attentional focus is associated with superior acute performance outcomes compared with an internal focus [50].

The RVC-normalised analysis yielded a larger standardised effect than the non-normalised sensitivity analysis. This most likely reflects reduced between-participant variability once EMST-related sEMG activity is expressed relative to each participant’s own cough-derived reference, since Cohen d depends not only on the mean difference but also on the variability of the difference scores. Visit-to-visit drift in the RVC denominator is unlikely to account for this difference. The reference value was on average slightly higher in the feedback condition, which would tend to attenuate rather than inflate the normalised feedback effect.

Consistent with evidence that visual feedback can stabilize within-session performance [41, 43, 51], participants maintained similar IO sEMG activation across all sets with SpiroGym feedback, with mean values remaining within 0% to − 5% of the first set. In contrast, IO sEMG activation declined across sets without feedback, with mean values decreasing by approximately − 8%, − 13%, − 12%, and − 16% relative to the first set. If this feedback-related maintenance of higher IO activation across sets is sustained across training sessions over a longer EMST program, this may increase the effective training dose, potentially helping to explain previously reported between-group differences in MEP training effects with versus without visual feedback [20]. Notably for clinical practice, when participants transitioned from the feedback to the no-feedback condition, IO activation decreased significantly. This transient effect of feedback has been reported consistently in the literature [21]. Therefore, to maximize EMST performance, ongoing feedback may be needed throughout the whole training period.

For secondary outcomes, visual feedback resulted in significantly higher EO sEMG activity during expiratory maneuvers (+ 21%) compared with EMST performed without feedback. No significant between-condition difference was observed in RA sEMG activity. This suggests that visual feedback enhances activation of the lateral abdominal wall, which plays a key role in forceful expiration, including MEP generation and cough [26, 42]. We also explored whether common neuropsychiatric symptoms (depression, apathy and anxiety) were associated with the magnitude of the acute feedback effect (ΔIO) [52]. Statistically significant associations between feedback responsiveness and depression, apathy, or anxiety were not detected in this exploratory analysis. Given the limited sample size, these findings should be considered hypothesis-generating. Another clinically relevant observation was derived from participants’ questionnaire responses. Participants reported no difficulty understanding the information displayed in the SpiroGym app, suggesting high usability of the feedback interface in this PD cohort. This is in line with previously published studies [17–19]. Moreover, most participants indicated that the SpiroGym visual feedback helped their EMST performance mainly due to increased motivation, with additional perceived benefits including support for maintaining consistent performance and reassurance about correct maneuver execution. This provides supportive evidence that the feedback format is understandable and perceived as beneficial, which is an important prerequisite for real-world implementation.

This study is not without limitations. First, the sample-size calculation was based on an internal pilot whose participants were also included in the final analysed sample. This approach may have introduced upward bias in the anticipated effect size and may have overstated the achieved statistical power. The study should therefore be interpreted as adequately powered to detect large within-participant effects, but not necessarily moderate ones, and the relatively wide 95% confidence interval around the primary effect should be interpreted in this context. In addition, the study was not powered to draw definitive conclusions regarding secondary endpoints. Findings related to EO and RA sEMG activity should therefore be considered hypothesis-generating and require confirmation in adequately powered studies. Second, although exploratory correlation analyses did not detect statistically significant associations between the magnitude of the acute visual-feedback effect and depressive symptoms, apathy, or anxiety in this sample, these findings should not be interpreted as evidence of absence of such relationships. They should be considered hypothesis-generating, given the limited sample size, limited power to detect moderate correlations, and generally low neuropsychiatric symptom severity in our cohort. Third, when interpreting the baseline participants clinical characteristics, it should be noted that baseline MEP differed between randomization sequences. However, EMST intensity was individualized to 75% of each participant’s own MEP, and both conditions were completed by each participant within the crossover design. This design feature reduced, although may not have completely eliminated, the potential impact of between-sequence differences. Fourth, although electrode placement followed standardized anatomical recommendations, complete separation of IO and EO activity cannot be guaranteed using surface EMG because of the anatomical proximity of these muscles and the potential for crosstalk. Fifth, MEP was measured only during the familiarisation session and was not re-measured at the two study visits, which were two weeks apart. Visit-to-visit drift in MEP therefore cannot be excluded, and if such drift occurred, the relative intensity of the EMST150 load may have differed between visits. This concern is mitigated by the standardisation of medication state and time of day across visits and by the randomisation of condition order, but it remains a limitation of the design. Finally, because each participant was exposed to the SpiroGym visual feedback within a single acute session, this crossover design cannot exclude a novelty or Hawthorne contribution to the observed effect. The acute increase in expiratory muscle activation should therefore not be extrapolated to the adaptations expected from long-term training.

Future studies should further clarify whether SpiroGym visual feedback affects other muscles involved in EMST, such as swallowing-related muscles. Adequately powered studies are also needed to determine which PwP benefit most from visual feedback (e.g., based on cognition, disease severity, or neuropsychiatric symptom burden).

## Conclusion

SpiroGym visual feedback during EMST acutely increased expiratory muscle activation in PwP, with higher IO sEMG amplitude maintained across all sets. In contrast, IO activation progressively declined across sets without visual feedback, which may explain previously reported between-group differences in MEP training effects when EMST is performed with versus without visual feedback.

## Supplementary Information

Below is the link to the electronic supplementary material.

Supplementary Material 1.

## Data Availability

We have deposited an open-access, de-identified participant-level dataset at  https://github.com/Neurorehab9/EXPARK.

## Abbreviations

BDI-II: Beck depression inventory-II
CI: Confidence interval
CONSORT: Consolidated Standards of Reporting Trials
EMST: Expiratory muscle strength training
EO: External oblique
ICC: Intraclass correlation coefficient
IO: Internal oblique
LEDD: Levodopa equivalent daily dose
MEP: Maximum expiratory pressure
mHealth: mobile health
MoCA: Montreal cognitive assessment
MVC: Maximal voluntary contraction
PD: Parkinson’s disease
PwP: People with Parkinson’s disease
RA: Rectus abdominis
RVC: Reference voluntary contraction
SAS: Starkstein apathy scale
sEMG: Surface electromyography
STAI: State-Trait Anxiety Inventory
TA: Transversus abdominis
MDS-UPDRS III: Movement Disorder Society–Unified Parkinson’s Disease Rating Scale Part III
VAFS: Visual analog fatigue scale
