# Supplementary material for: Visual feedback increases expiratory muscle training effort in Parkinson’s disease: a randomised crossover trial
Source: J Neuroeng Rehabil. 2026 Jul 22;23:298. doi: 10.1186/s12984-026-02104-5 (PMC13673806; doi:10.1186/s12984-026-02104-5)
Supplement: Supplementary file 1 [file 12984_2026_2104_MOESM1_ESM.docx]

**Supplementary Material**

**Supplementary Table 1.** Intra-session reliability of peak surface EMG amplitudes across the three voluntary cough trials for each recorded muscle at the two study visits.

| **Muscle** | **Visit** | **ICC(2,1)** | **95% CI** |
| --- | --- | --- | --- |
| Internal oblique (IO) | 1 | 0.905 | 0.868–0.933 |
| Internal oblique (IO) | 2 | 0.942 | 0.920–0.959 |
| External oblique (EO) | 1 | 0.874 | 0.828–0.910 |
| External oblique (EO) | 2 | 0.836 | 0.739–0.895 |
| Rectus abdominis (RA) | 1 | 0.932 | 0.906–0.952 |
| Rectus abdominis (RA) | 2 | 0.756 | 0.676–0.821 |

ICC(2,1), two-way random-effects, single-measurement, absolute-agreement intraclass correlation coefficient; CI, confidence interval; IO, internal oblique; EO, external oblique; RA, rectus abdominis.

**Supplementary Table 2.** Cough-derived reference voluntary contraction (RVC) peak surface EMG amplitudes (mV) for each recorded muscle at the two study visits, two weeks apart.

| **Muscle** | **Visit 1 (mean ± SD)** | **Visit 2 (mean ± SD)** |
| --- | --- | --- |
| Internal oblique (IO) | 0.060 ± 0.041 | 0.070 ± 0.048 |
| External oblique (EO) | 0.059 ± 0.028 | 0.063 ± 0.036 |
| Rectus abdominis (RA) | 0.037 ± 0.039 | 0.032 ± 0.024 |

Values are mean ± standard deviation of the peak sEMG amplitude across the three voluntary cough trials, in mV. The RVC obtained at each visit was used to normalise the EMST sEMG signals recorded during that same visit. SD, standard deviation; IO, internal oblique; EO, external oblique; RA, rectus abdominis.

**Supplementary Table 3.** Sensitivity analysis

The within-participant difference in mean peak non-normalized internal oblique sEMG activity during EMST performed with visual feedback was significantly greater than without feedback (mV; with feedback: 0.084 ± 0.062; without feedback: 0.056 ± 0.042; *p* < 0.0002; Cohen *d* = 0.48 [95% CI 0.16, 1.11].

|  | **No-FB, mean±SD (%)** | ***p-value***  ***vs Set 1*** | **FB, mean±SD (%)** | ***p-value***  ***vs Set 1*** | ***p*-value**  **(No-FB vs.FB)** |
| --- | --- | --- | --- | --- | --- |
| **1st set** | 0.06±0.05 | - | 0.09±0.07 | - | 0.003 |
| **2nd set** | 0.06±0.04 | 0.040 | 0.09±0.07 | 0.629 | 0.002 |
| **3rd set** | 0.05±0.04 | 0.006 | 0.08±0.05 | 0.119 | 0.001 |
| **4th set** | 0.06±0.04 | 0.020 | 0.08±0.06 | 0.210 | 0.002 |
| **5th set** | 0.05±0.04 | 0.015 | 0.08±0.06 | 0.396 | 0.001 |

Internal oblique surface electromyographic activity across sets during expiratory muscle strength training (5 maneuvers per set), normalized to reference voluntary contraction.

**Abbreviations:** No-FB = EMST without visual feedback; FB = EMST with visual feedback; p-value vs Set1 = within-condition comparison vs 1st set; p-value (No-FB vs. FB) = between-condition comparison. P values were adjusted for multiple comparisons.

**Supplementary Table 4.** Generalized linear mixed-effects model of normalised internal-oblique sEMG activity (%RVC). Gamma distribution, reciprocal link; coefficients are on the 1/%RVC scale. Model: IO ~ 1 + Sequence × Condition + (1 | ID); N = 38 (19 participants × 2 conditions).

| **Term** | **Estimate** | **SE** | **95% CI** | **p-value** |
| --- | --- | --- | --- | --- |
| Intercept | 0.010862 | 0.000916 | 0.0090 to 0.0127 | < 0.001 |
| Sequence | 0.000376 | 0.001283 | −0.0022 to 0.0030 | 0.772 |
| Condition (feedback vs no feedback) | −0.002665 | 0.001148 | −0.0050 to −0.00033 | 0.026 |
| Sequence × Condition (carryover) | 0.000138 | 0.001616 | −0.0031 to 0.0034 | 0.933 |

**Supplementary Table 5.** Internal oblique surface electromyographic activity by randomization arm.

|  | **Sequence:**  **Control-SpiroGym** | | | | | **Sequence:**  **SpiroGym-Control** | | | | | |
| --- | --- | --- | --- | --- | --- | --- | --- | --- | --- | --- | --- |
| **Protocol** | **n** | **Mean** | **SD** | **p-value** | **Cohen *d***  **[95% CI]** | **n** | **Mean** | **SD** | **p-value** | **Cohen *d***  **[95% CI]** |  |
| No-FB | 9 | 92.1 | 24.2 | 0.055 | 0.786  [-0.145 -1.695] | 10 | 89.0 | 19.6 | 0.002 | 1.175  [0.239-2.083] |  |
| FB | 9 | 122 | 45.2 |  |  | 10 | 114.8 | 22.4 |  |  |  |

Values are mean ± SD of normalised peak internal oblique sEMG activation (% RVC) during EMST, shown separately for each randomisation sequence. The p-value refers to the within-sequence between-condition comparison (EMST without visual feedback vs EMST with visual feedback), tested with the two-sided Wilcoxon signed-rank test. Cohen d (with 95% CI) refers to the same between-condition difference within each sequence. No-FB, EMST without visual feedback; FB, EMST with visual feedback; RVC, reference voluntary contraction; CI, confidence interval; SD, standard deviation.

**Supplementary Table 6** Correlations between the feedback-related change in normalized internal oblique surface EMG activation and neuropsychiatric symptom scores.

| **Correlation** | **r-value** | **p-value** |
| --- | --- | --- |
| ΔIO %RVC vs STAI (State) | 0.050 | 0.839 |
| ΔIO %RVC vs STAI (Trait) | 0.088 | 0.720 |
| ΔIO %RVC vs BDI-II | 0.109 | 0.657 |
| ΔIO %RVC vs SAS | 0.185 | 0.449 |

**Abbreviations:** ΔIO, within-participant difference in normalized internal oblique surface EMG activation (visual feedback − no feedback); STAI, State–Trait Anxiety Inventory; BDI-II, Beck Depression Inventory; SAS, Starkstein Apathy Scale.

**Supplementary Table 7.** Open-ended responses to the question: “To what degree does SpiroGym feedback help EMST performance?”

| **Respondent** | **Open-ended response** |
| --- | --- |
| **1** | Ambition—to reach a higher level |
| **2** | Motivation |
| **3** | Reassurance that the maneuver was performed correctly |
| **4** | You try harder when you have feedback |
| **5** | Motivation |
| **6** | Motivation |
| **7** | Motivation; effort to keep performance consistent |
| **8** | Motivation |
| **9** | I am already motivated to perform EMST, even without visual feedback |
| **10** | Motivation; effort to keep performance consistent |
| **11** | Motivation |
| **12** | It monitors the maneuver execution; it’s better |
| **13** | Motivation. Visual feedback makes me try harder |
| **14** | Effort to keep performance consistent |
| **15** | Motivation |
| **16** | I put in more effort and think more about the maneuver; I can see the result of the expiratory maneuver |
| **17** | The SpiroGym graph is instructive |
| **18** | I feel neutral about SpiroGym feedback |
| **19** | Motivation |

| **CONSORT checklist for randomised crossover trials** | | | |
| --- | --- | --- | --- |
| **Section/topic** | **Item No** | **Description** | **Page No** |
| Title† | 1a | Identification as a randomised crossover trial in the title | 1 |
| Abstract† | 1b | Specify a crossover design and report all information outlined in table 2 | 2 |
| Introduction: Background‡ | 2a | Scientific background and explanation of rationale | 3-4 |
| Introduction: Objectives‡ | 2b | Specific objectives or hypotheses | 3-4 |
| Methods: Trial design† | 3a | Rationale for a crossover design. Description of the design features including allocation ratio, especially the number and duration of periods, duration of washout period, and consideration of carry over effect | 13-16,20 |
| Methods: Change from protocol‡ | 3b | Important changes to methods after trial commencement (such as eligibility criteria), with reasons | n/a |
| Methods: Participants‡ | 4a | Eligibility criteria for participants | 14 |
| Methods: Settings and location‡ | 4b | Settings and locations where the data were collected | 13 |
| Methods: Interventions† | 5 | The interventions with sufficient details to allow replication, including how and when they were actually administered | 16-19 |
| Methods: Outcomes‡ | 6a | Completely defined prespecified primary and secondary outcome measures, including how and when they were assessed | 15-18 |
| Methods: Changes to outcomes‡ | 6b | Any changes to trial outcomes after the trial commenced, with reasons | n/a |
| Methods: Sample size† | 7a | How sample size was determined, accounting for within participant variability | 20-21 |
| Methods: Interim analyses and stopping guidelines‡ | 7b | When applicable, explanation of any interim analyses and stopping guidelines | 20-21 |
| Randomisation: Sequence generation‡ | 8a | Method used to generate the random allocation sequence | 14 |
| Randomisation: Sequence generation‡ | 8b | Type of randomisation; details of any restriction (such as blocking and block size) | 14 |
| Randomisation: Allocation concealment mechanism‡ | 9 | Mechanism used to implement the random allocation sequence§ (such as sequentially numbered containers), describing any steps taken to conceal the sequence until interventions were assigned | 14 |
| Randomisation: Implementation† | 10 | Who generated the random allocation sequence,§ who enrolled participants, and who assigned participants to the sequence of interventions | 14 |
| Blinding‡ | 11a | If done, who was blinded after assignment to interventions (for example, participants, care providers, those assessing outcomes) and how | 14 |
| Blinding: Similarity of interventions‡ | 11b | If relevant, description of the similarity of interventions | 14 |
| Statistical methods† | 12a | Statistical methods used to compare groups for primary and secondary outcomes which are appropriate for crossover design (that is, based on within participant comparison) | 20-21 |
| Statistical methods: Additional analyses‡ | 12b | Methods for additional analyses, such as subgroup analyses and adjusted analyses | 20-21 |
| Results: Participant flow (a diagram is strongly recommended)† | 13a | The numbers of participants who were randomly assigned, received intended treatment, and were analysed for the primary outcome, separately for each sequence and period | 7 |
| Results: Losses and exclusions† | 13b | No of participants excluded at each stage, with reasons, separately for each sequence and period | 7 |
| Results: Recruitment‡ | 14a | Dates defining the periods of recruitment and follow-up | 13-14 |
| Results: Trial end‡ | 14b | Why the trial ended or was stopped | n/a |
| Results: Baseline data† | 15 | A table showing baseline demographic and clinical characteristics by sequence and period | 6 |
| Results: Numbers analysed† | 16 | Number of participants (denominator) included in each analysis and whether the analysis was by original assigned groups | 7 |
| Results: Outcomes and estimation† | 17a | For each primary and secondary outcome, results including estimated effect size and its precision (such as 95% confidence interval) should be based on within participant comparisons.¶ In addition, results for each intervention in each period are recommended | 4-5 |
| Results: Binary outcomes‡ | 17b | For binary outcomes, presentation of both absolute and relative effect sizes is recommended | 4-5 |
| Results: Ancillary analyses‡ | 18 | Results of any other analyses performed, including subgroup analyses and adjusted analyses, distinguishing prespecified from exploratory | 4-5 |
| Results: Harms† | 19 | Describe all important harms or untended effects in a way that accounts for the design (for specific guidance, see CONSORT for harms32) | 4 |
| Discussion: Limitations† | 20 | Trial limitations, addressing sources of potential bias, imprecision, and if relevant, multiplicity of analyses. Consider potential carry over effects | 12-13 |
| Discussion: Generalisability‡ | 21 | Generalisability (external validity, applicability) of the trial findings | 13 |
| Discussion: Interpretation‡ | 22 | Interpretation consistent with results, balancing benefits and harms, and considering other relevant evidence | 11-13 |
| Other information: Registration‡ | 23 | Registration number and name of trial registry | 2,13 |
| Other information: Protocol‡ | 24 | Where the full trial protocol can be accessed, if available | n/a |
| Other information: Funding‡ | 25 | Sources of funding and other support (such as supply of drugs), role of funders | 22 |
